# Separation and determination of estrogen in the water environment by high performance liquid chromatography-fourier transform infrared spectroscopy

**DOI:** 10.1038/srep32264

**Published:** 2016-08-31

**Authors:** Bei Zheng, Wentao Li, Hongyan Li, Lin Liu, Pei Lei, Xiaopeng Ge, Zhiyong Yu, Yiqi Zhou

**Affiliations:** 1 Key Laboratory of Drinking Water Science and Technology, Chinese Academy of Sciences, Beijing 100085, China; 2 Northern Engineering Design and Research International Co., Shijiazhuang 050011, China; 3 Research Center for Eco-Environment Sciences, Chinese Academy of Sciences, Beijing 100085, China

## Abstract

The components for connecting high-performance liquid chromatography (HPLC) with Fourier-transform infrared spectroscopy (FTIR) were investigated to determine estrogen in the water environment, including heating for atomization, solvent removal, sample deposition, drive control, spectrum collection, chip swap, cleaning and drying. Results showed that when the atomization temperature was increased to 388 K, the interference of mobile phase components (methanol, H_2_O, acetonitrile, and NaH_2_PO_4_) were completely removed in the IR measurement of estrogen, with 0.999 of similarity between IR spectra obtained after separation and corresponding to the standard IR spectra. In experiments with varying HPLC injection volumes, high similarity for IR spectra was obtained at 20 ul injection volume at 0.01 mg/L BPA while a useful IR spectrum for 10 ng/L BPA was obtained at 80 ul injection volume. In addition, estrogen concentrations in the natural water samples were calculated semi-quantitatively from the peak intensities of IR spectrum in the mid-infrared region.

High performance liquid chromatography (HPLC) is an important way to analyze organic compounds with high boiling point and high molecular weight^1^,^2^, but it cannot determine quantitatively the unknown component or structure^3^. Fourier transform infrared spectrometry (FTIR) has been considered a fingerprint identification technology since the birth of FTIR. A compound with the ability of absorbing in the infrared light will produce a characteristic IR spectrum, because of FTIR unique discrimination ability. IR has been widely applied in the environmental science research field, for example in adsorption^4^,^5^, online-redox reaction analysis^6^, and especially in the determination of organic compounds in the aquatic environment^7^,^8^. However, the *in-situ* IR technology has been limited in the analysis of organic compounds in aquatic environments, due to the interference of water molecular absorption and the limitation of separation technology^9^. Fortunately, the Attenuated Total Reflection (ATR) technology can overcome these defects of FTIR and realize *in-situ* detection the solid-liquid interface reactions^10^. The coupling of ATR-FTIR and chromatographic techniques with high separation capability can overcome many shortcomings of chromatographic analysis, to obtain the IR spectrum of individual components in a mixture. Although the coupling of gas chromatography (GC) and different types of FTIR has successfully been identified each component from mixtures^11^,^12^,^13^,^14^, organic compounds with high molecular weight and high boiling point were separated difficultly by GC. Therefore, GC-FTIR is seriously limited in the application^15^,^16^,^17^,^18^.

In addition, chromatography-spectroscopy technology has been focused on HPLC-FTIR because of the unique advantage of HPLC^7^,^19^,^20^,^21^,^22^. The key issue of HPLC-FTIR is how to connect HPLC to FTIR, and to eliminate the interference of the mobile phase and solvent. IR characteristic peaks of target contaminants are masked in a mobile phase with strong infrared adsorption. Although some methods were reported to solve this difficult problem in the 1990s, such as flow extraction^23^,^24^, chemical reaction^25^, metal wire^26^, and continuous spray^27^, each of these methods had some defects needing further modification. For example, the disadvantage of flow extraction and chemical reaction is not to eliminate the interference of water and organic solvents in the IR measurement of the target contaminants; the defects of metal wire is only to collect a part of sample amount; the shortcoming of continuous spray is to the lower signal-to-noise ratio.

Based on the above reasons, the coupling of ATR-FTIR and HPLC could improve the signal-to-noise ratio. Heating under N_2_ atmosphere was introduced to decrease the interference of mobile phase in the IR measurement of the target contaminants. Components of a ZnSe crystal sample holder, rotating platform and high heat atomizer were introduced to compose a new HPLC-FTIR connection model. Using this platform, the common contaminant estrogen was analyzed to investigate the separation and determination performance of HPLC-FTIR technology.

## Results and Discussion

### Mobile phase removal

The components of the HPLC mobile phase, including methanol, acetonitrile, NaH_2_PO_4_, and H_2_O, absorb in the infrared light, which interferes with the IR measurement of the target contaminants. Even though the organic components in the mobile phase were evaporated by N_2_ blowing alone, water molecules were difficult to remove completely in a large fraction of the mobile phase. The three solvents (methanol, acetonitrile, and water) had different boiling points, as shown in Table S1, so heating under N_2_ atmosphere was introduced to enhance the effect of blowing N_2_. In the measurements, four temperatures (298 K, 348 K, 368 K, 388 K) were chosen to investigate mobile phase removal. IR spectra of methanol, acetonitrile, NaH_2_PO_4_, and H_2_O were obtained by ATR-FTIR, and were used as standard IR spectra (Fig. S2). Meanwhile, the characteristic peaks in the standard spectra were labeled, as summarized in Table 1. These characteristic peaks were used to indicate whether the mobile phase was removed completely or not.

Figure S3 shows the IR spectra of the mobile phase obtained by passage through the HPLC-FTIR connection component at different temperatures, which are summarized in Table 2. As shown in Fig. S3a, there were 13 IR characteristic peaks of the mobile phase at 298 K, such as 875.52 cm^−1^, 933.64 cm^−1^, 1290.00 cm^−1^ for NaH_2_PO_4_; 749.31 cm^−1^, 1376.23 cm^−1^, 2253.71 cm^−1^, and 2293.35 cm^−1^ for acetonitrile; 1028.85 cm^−1^, 1449.08 cm^−1^, 2832.46 cm^−1^, and 2944.75 cm^−1^ for methanol; 1647.27 cm^−1^ and 3424.18 cm^−1^ for H_2_O. At 348 K, 12 IR characteristic peaks were detected (Fig. S3b), including 1646.93 cm^−1^ and 3424.79 cm^−1^ for H_2_O and residual peaks for acetonitrile, whereas the IR peaks of methanol and NaH_2_PO_4_ disappeared. When the temperature of the heating pipe was increased to 368 K, only the IR peaks of H_2_O were left, for instance, 699.59 cm^−1^, 1647.87 cm^−1^, and 3424.49 cm^−1^ (Fig. S3c). All IR peaks disappeared at 388 K (Fig. S3d), which indicated that the mobile phase was removed completely. The optimal temperature was 388 K.

### Separation and determination of individual contaminant

The interference of the mobile phase was avoided efficiently by controlling the temperature under N_2_ atmosphere. To obtain good IR spectra of individual contaminant, separation and determination was further investigated from a mixture of individual contaminant and mobile phase. In the experiments, five estrogen compounds at 0.01 mg/L were mixed with the mobile phase, respectively. IR spectra were obtained under different separation conditions, as shown in Fig. S4.

**Figure 1:**
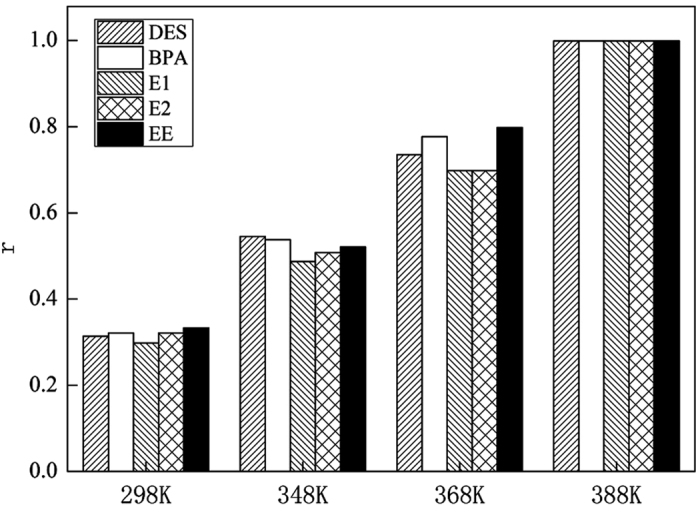
Correlation coefficients for five estrogen compounds (DES, E1, E2, EE, BPA) at different temperatures (298 K, 348 K, 368 K, 388 K).

Figure 1 shows the correlation coefficient r for the five estrogen compounds at different temperatures. With the increase of separation temperature, characteristic peaks of methanol, NaH_2_PO_4_, acetonitrile and H_2_O disappeared, leading to the highlighting of sample information. Thus the similarity of the obtained and standard spectrum was increased. As shown in Fig. 1, the correlation coefficient r was increased from 0.3 to 0.999 with the increase of heating temperature, indicating that the obtained IR spectra were consistent with standard IR spectra at 388 K. The mixture of individual contaminant and mobile phase was efficiently separated and the contaminants could be determined through HPLC-FTIR technology.

### Separation and determination of multiple contaminants

**Figure 2:**
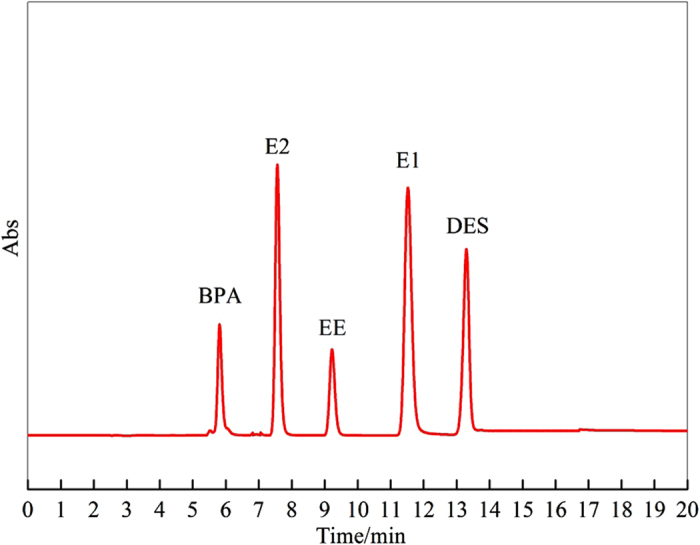
HPLC diagram for five estrogen compounds of DES, E1, E2, EE, BPA.

**Figure 3:**
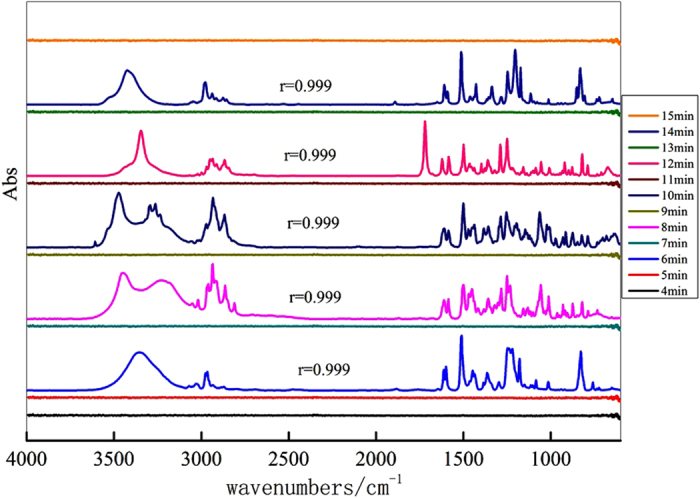
IR spectra of the five estrogen compounds obtained at different acquisition time via HPLC-FTIR interface module: BPA-6 min; E2-8 min; EE-10 min; E1-12 min: DES-14 min.

To further investigate the separation and determination ability for multiple contaminants via HPLC-FTIR, a mixture of five estrogen compounds at 0.01 mg/L each was chosen to carry out the separation and determination experiments. As shown in Fig. 2, the peak time of BPA, E2, EE, E1, and DES was 5.83 min, 7.56 min, 9.23 min, 11.54 min, and 13.30 min, respectively. Based on the peak time for HPLC, IR signal collection began at 4 min. The interval time for subsequent data collection was set at 1 min. Results (Fig. 3) showed that IR spectra were obtained at the different collection time. IR spectra were obtained at 6 min, 8 min, 10 min, 12 min, and 14 min, while no IR signal was observed for the rest of the collection time. The standard IR spectra of the five estrogen compounds were almost the same as the IR spectra obtained at different collection time, with correlation coefficients of not less than 0.999. This showed that the identification information obtained from IR spectra at different collection time agreed well with the separation information from HPLC, indicating that HPLC-FTIR connection component was effective for determination of estrogen compounds.

### Optimization of injection conditions

In the HPLC-FTIR technology, the injection volume was critical for the sensitivity of separation and determination of the target contaminants. Low injection volume can reduce the sensitivity of IR detection with a decrease in the signal-to-noise ratio, while high injection volume can contaminate the chromatographic column, leading to weak separation between chromatographic components^28^. To obtain the optimized injection conditions, 0.01 mg/L BPA and different injection volumes (5, 10, 20, 30, 50, and 80 μL) were chosen to investigate similarity between the standard IR spectrum and the obtained IR spectrum.

**Figure 4:**
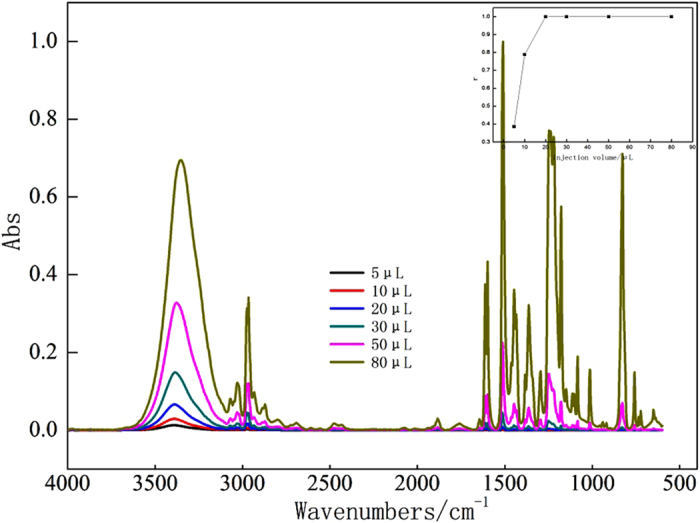
IR spectrums of BPA and similarity under different injection volume (5–80 ul).

As shown in Fig. 4, the peak intensity and area of BPA in the IR spectrum increased with the increase of injection volume. The IR signal was quite weak at 5 μl injection volume, giving a correlation coefficient of 0.386. When the injection volume was 80 μl, the peak area and intensity reached their maximum. Regarding to the relation between similarity and injection volume, r was as high as 0.999 at 20 μl injection volume. Although other parameter values of the IR spectrum obtained at 20 μl were lower than those at 50 μl or 80 μl, the IR spectrum obtained was quite useful. Therefore, 20 μl of injection volume was chosen to prolong column lifetime and decrease the risk of error that a contaminated column brought. If the concentration of contaminant was quite low, the measurement sensitivity was improved by increasing the injection volume.

### Precision, stability and detection limit of HPLC-FTIR

Precision, stability and sensitivity are quite important in the application of HPLC-FTIR. Choosing BPA as the target contaminant, RSD was calculated based on five repeated measurements to evaluate the precision of HPLC-FTIR. For stability, BPA was determined at 0 h, 4 h, 8 h, 16 h, and 24 h after separation, to calculate RSD. Different concentration (0.1–0.00001 mg/L) of BPA was analyzed to evaluate the detection limit of HPLC-FTIR. The injection volume of low concentration sample was 80 μl to improve sensitivity.

In Table S2, the RSD values of 22 characteristic peaks were less than 0.1%, which were calculated from five duplicate measurements. With the increase of measuring time, RSD values were less than 0.1% (Table S3). These results satisfied the analytical chemical requirements of precision and stability^29^.

**Figure 5:**
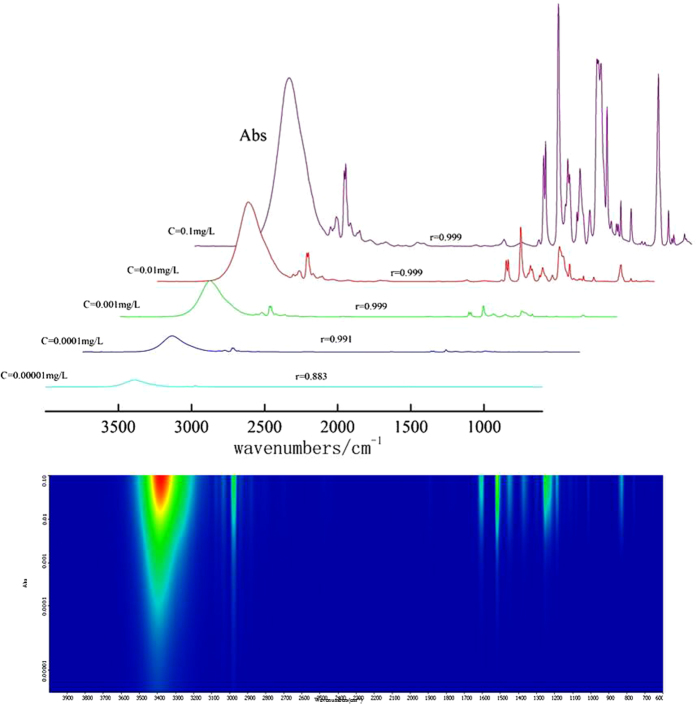
Three-dimensional diagrams of IR spectra of different BPA concentration captured via HPLC-FTIR interface module.

**Figure 6:**
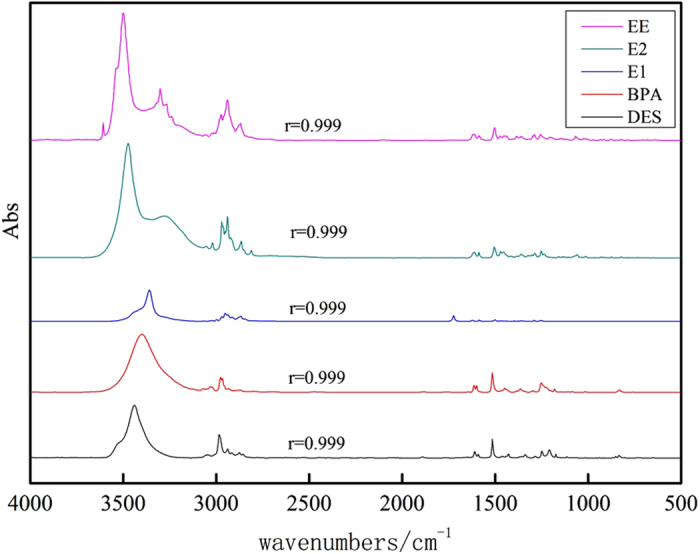
IR spectrums for five estrogen compounds of water sample (Yangtze River) after 0.001 mgL of five estrogen compounds mixture added.

Figure 5 shows 3D plots of IR spectra obtained at different concentrations of BPA. Color represents the IR signal intensity, for instance, the IR signal intensity is weaker as the color changes from red to yellow, green, sky blue, and dark blue. As shown in Fig. 6, the IR signal captured became stronger from 0.001 mg/L to 0.1 mg/L, when r between the standard IR spectrum and obtained IR spectrum was more than 0.999. For 0.0001–0.00001 mg/L BPA, r was 0.991-0.883. IR peak intensity decreased apparently between 600 cm^−1^ and 1800 cm^−1^, while the IR signal was quite strong from 2000 cm^−1^ to 3500 cm^−1^. Although r declined a bit, BPA was still identified, indicating that HPLC-FTIR was suitable to analyze low concentrations.

### Analysis and semi-quantitative calculation of estrogen compounds in natural water

Actual water from the Yellow River, Yangtze River, Liao River, Songhua River, Hai River, and Tai Lake was analyzed using HPLC-FTIR, and the results were compared with those of HPLC-UV. The five estrogen compounds were not detected in the six water basins through HPLC-FTIR, and this result agreed with HPLC-UV. To investigate the advantages of HPLC-FTIR, a mixture containing 0.001 mg/L of the five estrogen compounds was added to the water sample from the Yangtze River, which was then measured by HPLC-FTIR and HPLC-UV. The five estrogen compounds were detected by HPLC-FTIR instead of HPLC-UV, because the detection limit of HPLC-UV was higher than 0.001 mg/L. Although the peak intensity at low wavenumbers decreased slightly in the IR spectrum, r remained constant at 0.999. The sensitivity of HPLC-FTIR was higher than that of HPLV-UV, indicating that HPLC-FTIR has quite significant value in water quality analysis.

To investigate the estrogen level in the natural water samples, the intensities of IR characteristic peaks that were obtained via the HPLC-FTIR technology were used to calculate contaminant concentration, as previously reported^30^. First, the best characteristic peak was chosen because of a number of IR peaks in the pure material. The IR spectrum of BPA was divided into a high wavenumber band (2000–3000 cm^−1^), a mid wavenumber band (1200–1300 cm^−1^, 1500–1700 cm^−1^) and a low wavenumber band (700–800 cm^−1^), where linear relationships were obtained between the BPA concentration (0.0001-0.01 mg/L) and absorbance of different wavenumber bands as shown in Figs S5 and S6. In Table 3, the linear correlation coefficient (R^2^) was up to 0.999 in the mid IR band (1200–1300 cm^−1^, 1500–1700 cm^−1^) while R^2^ was lower in the low or high wavenumber bands. Therefore, the mid wavenumber band was chosen to calculate the contaminant concentration. Secondly, standard curves of the five estrogen compounds were prepared as shown in Fig. S8, including wavenumber selection of the five estrogen compounds shown in Fig. S7. Linear equation parameters are listed in the Table 4, which show that good linear relations were obtained. Thirdly, a mixture of five estrogen compounds at 0.03 mg/L each was added to a water sample (Yangtze River) to verify the semi-quantitative performance of HPLC-FTIR. As shown in Table S4, the measured RSD values of estrogen concentrations were controlled within 6%, indicating that this method was feasible for measurement of estrogen levels via HPLC-FTIR.

## Experimental Section

### Instruments and Reagents

The HPLC (Thermo Fisher Ultimate 3000, USA) system consisted of a ternary gradient pump (flow rate: 2 ml/min), injection valve (injection volume: 100 μl), UV detector (wavelength: 230 nm) and analytical column (250 mm × 4.6 mm, INERTSUSTAIN C18). The gradient elution was performed with methanol (solvent A), acetonitrile (solvent B), and 10 mmol/L NaH_2_PO_4_ solution (solvent C). The gradient program was as follows: 0–13 min, 14:43:43 (solvent A/solvent B/solvent C, volume fraction); 13.01–19 min, 45:43:12 (solvent A/solvent B/solvent C, volume fraction); then the ratios of solvent A, solvent B and solvent C were changed to 14%, 43% and 43% in 2 min, respectively and kept for 3 min to equilibrate column.

FTIR measurements were performed on a Thermo Fisher Nicolet 8700 ATR- FTIR spectrometer equipped with a liquid nitrogen-cooled MCT detector (Hg-Cd-Te semiconductor film, as a photoconduction detector), which gives three orders of magnitudeimprovement in sensitivity compared to a DTGS detector and has been used in GC-FTIR technology^31^. All spectra were collected at the range of 600–4000 cm^−1^ using a resolution of 4 cm^−1^ and were averaged from 32 scans.

Diethylstilbestrol (DES, >99.9%), oestrone (E1, >99.9%), ethinylestradiol (EE, >99.9%), bisphenol A (BPA, >99.9%), and estradiol (E2, >99.9%) were purchased from Tokyo Kasei Kogyo Co., Ltd; Potassium bromide (KBr, chromatographically pure), methanol (CH_3_OH, PR), acetonitrile (CH_3_CN, PR), dichloromethane (CH_2_Cl_2_, PR) were obtained from Thermo Fisher Scientific; disodium hydrogen phosphate (NaH_2_PO_4_, AR) was purchased from Sinopharm Chemical Reagent Co., Ltd; the OA-SIS HLB SPE column was obtained from Water Co., Ltd.

### HPLC-FTIR connection component

**Figure 7:**
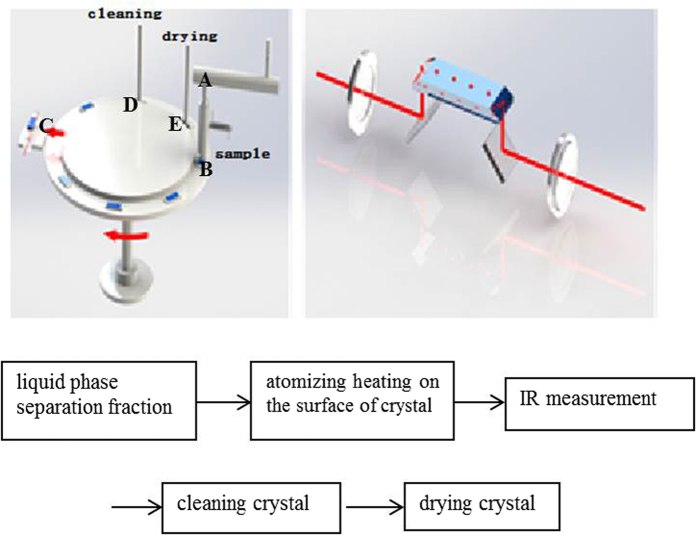
Coupling HPLC-FTIR interface module components and the flowchart of analysis.

The HPLC-FTIR connection component was located between the HPLC and FTIR (Fig. S1). As shown in Fig. 7, the HPLC-FTIR connection component was composed of an optical platform, ZnSe crystal sample holder, circular driving and rotating platform, heater sleeve, atomizer, cleaning and drying equipment. The operating procedure of the HPLC-FTIR connection component was as follows:

(1) mobile phase removal: target contaminants and mobile phase separated by the chromatographic column passed through the heater sleeve (A) under N2, where the mobile phase was removed at different temperatures (273–388 K).

(2) sample deposition: a single sample from the atomizer was scattered onto the surface of the ZnSe crystal (B).

(3) collection of IR spectrum: the ZnSe crystal was moved to the optical platform (C) by a circular driving and rotating platform, where the IR spectrum was collected. In the optical platform, two aluminum mirrors were set to reflect infrared light, which entered into the ZnSe crystal. Multiple reflections of the infrared light in the ZnSe crystal resulted in more contact between the light and the sample on the surface of the ZnSe crystal, producing more spectroscopic information.

(4) crystal cleaning: the ZnSe crystal used was sent to a cleaning unit (D), where it was washed well by absolute ethyl alcohol drawn by a pump, to avoid any interference with the next measurement.

(5) crystal drying: the clean ZnSe crystal was sent to a drying unit (E), where it was blown dry at high temperature.

The above operation was controlled with a circular driving and rotating platform, to realize on-line monitoring of the batch samples.

## Methods

Single solutions: 0.1 mg/ml DES, E1, EE, BPA, and E2 were dissolved in methanol as single stock solutions, respectively. Mixed solution: 0.1 mg/ml of DES, E1, EE, BPA and E2 were dissolved in methanol as a mixed stock solution. In the experiments, the solutions were diluted to 0.1 mg/L. All the stock solutions were stored at 269 K.

In the experiments, the target contaminants were separated through HPLC and entered into the HPLC-FTIR connection component to remove the mobile phase, and then their IR spectra were collected via ATR-FTIR. The correlation coefficient was calculated between the obtained IR spectrum and standard IR spectrum, which was used to evaluate the separation and determination performance of the HPLC-FTIR connection component in the individual contaminant analysis^32^. Methods have been reported to calculate the similarity of spectra, such as related coefficient, angle cosine, and distance coefficient^33^. The related coefficient method was used to calculate similarity according to equation (1)^29^,^34^:


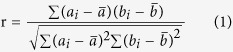
In equation (1), a_i_ and b_i_ are the corresponding values for the obtained IR spectrum and standard IR spectrum, respectively, where 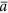 and 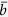 are average values. The correlation coefficient r was approximately 1.0, indicating that similarity was quite high. In view of the precision and stability of the IR spectra, two spectra were considered to be consistent when similarity was 0.999 or higher. Otherwise, they were considered to be different^29^. Meanwhile, standard IR spectra of five estrogen compounds were obtained by the KBr pressed disc technique to assure the reliability of the combined technologies.

The separation and determination performance of HPLC-FTIR technology was evaluated, including three main aspects: mobile phase removal, separation and determination of a single contaminant and mobile phase, and separation and determination of multiple contaminants and mobile phase. Based on the above results, the injection volume was further optimized to measure the precision, stability and detection limit of HPLC-FTIR technology.

To further investigate that the organic contaminants in the actual water environment were analyzed via HPLC-FTIR technique, water samples from the Yellow River, Yangtze River, Liao River, Songhua River, Hai River, and Tai Lake were filtered with a 0.45 μm membrane to remove solids before analysis, extracted via a SPE column activated with 10 ml methanol and 10 ml deionized water (pH 2–3) in sequence, and eluted by 10 ml CH_2_Cl_2_- methanol (80:20, V/V). The eluate was dried under N_2_, and diluted with methanol to 1 ml. Finally, samples were determined using HPLC-UV and HPLC-FTIR, respectively.

## Additional Information

**How to cite this article**: Zheng, B. *et al*. Separation and determination of estrogen in the water environment by high performance liquid chromatography-fourier transform infrared spectroscopy. *Sci. Rep.* **6**, 32264; doi: 10.1038/srep32264 (2016).

## Supplementary Material

Supplementary Information

## Figures and Tables

**Table 1:** Characteristic peaks of IR standard spectra for three different HPLC solvents and NaH_2_PO_4_.

Mobile phase	Characteristic Peaks (cm^−1^)				
methanol	663.62	1029.39	1115.32	1449.9	2044.68
	(γ_-CHO_)	—	—	(δas _-CH3_)	—
	2522.34	2832.48	2944.75	3341.19	
	—	(ν_-CH3_)	(ν_-CH3_)	(ν_-OH_)	
acetonitrile	749.31	918.88	1039.25	1376.03	1446.51
	—	—	—	(δs_-CH3_)	(δas _-CH3_)
	2252.43	2292.35	2944.08	3002.76	3164.29
	(ν_-CN_)	—	(ν_-CH3_)	—	—
H_2_O	699.76	1646.77	2140.01	3424.1	
	(ρ_H2O_)	(δ_H2O_)	—	(ν_H2O_)	
NaH_2_PO_4_	874.43	934.39	991.36	1060.21	1130.45
	—	(ν_P-OH_)	(ν_P-OH_)	(νas_-PO4_)	(νs_-PO2_)
	1165.18	1287.51	2399.59	2782.90	
	—	(νas_-PO2_)	(ν_-PH_)	(νP-OH)	

ν: stretching vibration δas: asymmetrical deformation vibration γ: out-plane flexural vibration.

δs: symmetrical deformation vibration ρ: rocking vibration δ: deformation vibration.

νas: asymmetric stretching vibration νs: symmetrical stretching vibration.

**Table 2:** Characteristic peaks of IR spectra of mobile phase at different temperatures.

Mixture	Characteristic Peaks (cm^−1^)						
298 K mobile phase	749.31	875.52	933.64	1028.85	1290.00	1376.23	1449.08
	1647.27	2253.71	2293.35	2832.46	2944.75	3424.18	
348 K mobile phase	750.12	918.25	1040.19	1377.27	1446.91	1646.93	2253.42
	2292.06	2944.06	3002.42	3164.35	3424.79		
368 K mobile phase	699.59	1647.87	3424.49				
388 K mobile phase	—	—	—	—	—	—	—

“—” means undetected.

**Table 3:** Parameters of linear fit curves under different wavenumbers.

Wavenumber (cm^−1^)	Parameters of linear fit curve		
	Intercept	Slope	R^2^
700–800	−0.0266	72.7993	0.9919
1200–1300	0.0017	30.2344	0.9997
1500–1700	0.0004	30.6556	0.9998
2900–3000	0.1278	27.3711	0.9090

**Table 4:** Fitting parameters of standard curves of DES, E1, E2, EE and BPA at near infrared region.

Samples	Fitting parameters		
	Intercept	Slope	R^2^
DES	0.0195	36.1278	0.9997
E1	0.0167	25.0473	0.9998
E2	−0.0006	33.4343	0.9995
EE	0.0010	30.1671	0.9997
BPA	0.0004	30.6556	0.9998
