# Supplementary material for: Separation and determination of estrogen in the water environment by high performance liquid chromatography-fourier transform infrared spectroscopy
Source: Sci Rep. 2016 Aug 31;6:32264. doi: 10.1038/srep32264 (PMC5006044; doi:10.1038/srep32264)
Supplement: Supplementary Information [file srep32264-s1.doc]

**Separation and Determination of** **estrogen in the water environment**

**by High Performance Liquid Chromatography-Fourier Transform**

**Infrared Spectroscopy**

Bei Zheng^1^, Wentao Li^1^, Hongyan Li^1^, Lin Liu^3^, Pei Lei^1^, Xiaopeng Ge^2^, Zhiyong Yu^1^, Yiqi Zhou^2*^

^1^Key Laboratory of Drinking Water Science and Technology, Chinese Academy of Sciences, Beijing 100085, China

^2^ Research Center for Eco-Environment Sciences, Chinese Academy of Sciences, Beijing 100085, China

^3^ Northern Engineering Design and Research International Co., Shijiazhuang 050011, China


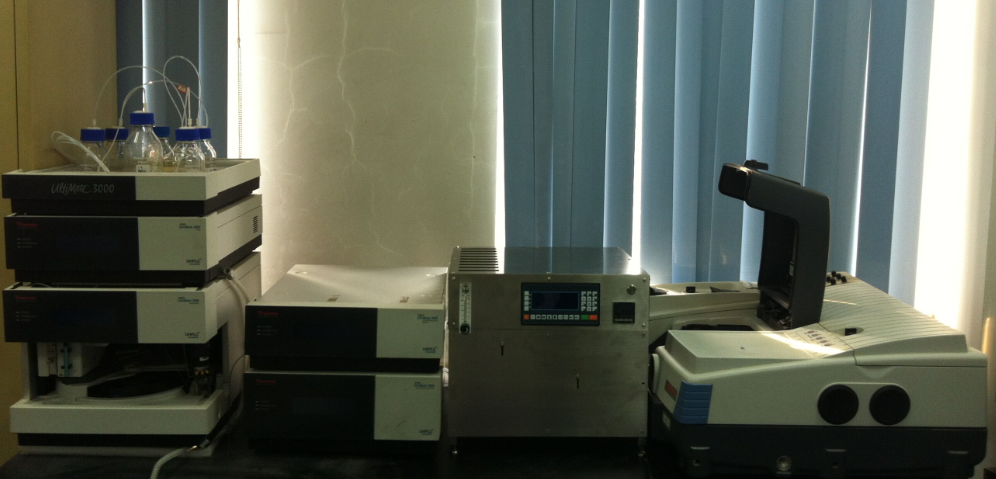


Figure S1 Image of coupling HPLC-FTIR device

Figure S2 Standard spectra of IR for the three different components in HPLC mobile phase:

a, methanol; b, H_2_O; c, acetonitrile; d, NaH_2_PO_4_

Figure S3 IR spectra of mobile phase at different temperatures：(a) 298 K, (b) 348 K, (c) 368 K, (d)388 K

Figure S4 IR spectra of five estrogen compounds after solvent removal at different temperatures:

(a) DES, (b) E1, (c) E2, (d) EE, (e) BPA

Figure S5. Wavenumber selection of BPA curves under different wavenumber bands:

a, 700-800cm^-1^; b, 1200-1300cm^-1^; c, 1500-1700 cm^-1^; d,2900-3000 cm^-1^

Figure S6. Standard linear fit curve of BPA concentration under different wavenumbers

Figure S7.Wavenumber selection of estrogen under near IR light region: a, DES; b, E1; c, E2; d, EE; e, BPA

Figure S8. Standard curves of DES, E1, E2, EE and BPA under near IR light region

Table S1. Boiling point of three different solvents commonly applied in the HPL C mobile phase

| Mobile phase | Boiling point |
| --- | --- |
| methanol | 337.7 K |
| acetonitrile | 353-355 K |
| water | 373 K |

Table S2. Precision of characteristic peaks of IR spectra of BPA for five duplicated measurements and calculated RSD values

| First time | Second time | Third time | Fourth time | Fifth time | average | RSD % |
| --- | --- | --- | --- | --- | --- | --- |
| 649.24 | 649.37 | 649.28 | 650.35 | 649.28 | 649.50 | 0.07 |
| 722.66 | 722.85 | 722.41 | 722.97 | 723.14 | 722.81 | 0.04 |
| 758.55 | 758.36 | 759.41 | 759.12 | 758.65 | 758.82 | 0.06 |
| 826.95 | 826.75 | 825.98 | 825.98 | 825.01 | 826.13 | 0.09 |
| 1013.62 | 1013.7 | 1013.8 | 1013.25 | 1013.76 | 1013.62 | 0.02 |
| 1084.06 | 1084.1 | 1085 | 1084.32 | 1084.18 | 1084.32 | 0.04 |
| 1113.49 | 1113.6 | 1113.8 | 1113.85 | 1113.77 | 1113.69 | 0.01 |
| 1178.31 | 1178.3 | 1178.6 | 1179.15 | 1180.1 | 1178.89 | 0.06 |
| 1219.96 | 1220.8 | 1221.9 | 1221.58 | 1222.5 | 1221.35 | 0.08 |
| 1246.71 | 1246.9 | 1245.2 | 1246.82 | 1246.17 | 1246.34 | 0.06 |
| 1296.26 | 1296.8 | 1298.5 | 1297.54 | 1297.51 | 1297.32 | 0.07 |
| 1363.02 | 1363.5 | 1363.9 | 1363.17 | 1361.15 | 1362.95 | 0.08 |
| 1384.29 | 1384.3 | 1384.5 | 1384.75 | 1383.1 | 1384.19 | 0.05 |
| 1435.26 | 1435.8 | 1435.9 | 1435.71 | 1435.26 | 1435.57 | 0.02 |
| 1446.58 | 1446.2 | 1446.6 | 1446.35 | 1448.12 | 1446.76 | 0.05 |
| 1510.56 | 1510.2 | 1510.3 | 1510.35 | 1510.35 | 1510.34 | 0.01 |
| 1598.71 | 1598.6 | 1598.8 | 1598.17 | 1599.77 | 1598.80 | 0.04 |
| 1611.91 | 1611.8 | 1611.7 | 1612.1 | 1612.51 | 1612.01 | 0.02 |
| 2965.87 | 2965.8 | 2965.9 | 2968.73 | 2965.54 | 2966.35 | 0.05 |
| 2975.37 | 2975.4 | 2975.8 | 2975.36 | 2975.65 | 2975.51 | 0.01 |
| 3028.79 | 3028.9 | 3029.5 | 3030.17 | 3029.14 | 3029.30 | 0.02 |
| 3349.34 | 3350.2 | 3350.8 | 3351.25 | 3350.17 | 3350.35 | 0.02 |

Table S3. Reproducibility stability for characteristic peaks of IR spectra of BPA measured at different time and calculated RSD values

| 0h | 4h | 8h | 16h | 24h | average | RSD % |
| --- | --- | --- | --- | --- | --- | --- |
| 649.37 | 649.4 | 649.15 | 650.17 | 649.74 | 649.57 | 0.06 |
| 722.66 | 722.84 | 722.57 | 722.11 | 723.54 | 722.74 | 0.07 |
| 758.14 | 758.68 | 758.71 | 758.17 | 758.68 | 758.48 | 0.04 |
| 826.08 | 826.75 | 826.17 | 825.99 | 826.15 | 826.23 | 0.04 |
| 1013.62 | 1013.2 | 1013.1 | 1013.57 | 1013.88 | 1013.46 | 0.03 |
| 1084.56 | 1084.8 | 1084.7 | 1084.5 | 1085.02 | 1084.71 | 0.02 |
| 1113.56 | 1113.6 | 1113.8 | 1113.58 | 1114.11 | 1113.73 | 0.02 |
| 1178.51 | 1178.5 | 1178.6 | 1178.95 | 1180.15 | 1178.95 | 0.06 |
| 1219.9 | 1220.6 | 1221.6 | 1221.58 | 1222.5 | 1221.23 | 0.08 |
| 1246.97 | 1246.6 | 1247.1 | 1246.98 | 1246.58 | 1246.83 | 0.02 |
| 1296.53 | 1296.8 | 1298.8 | 1296.65 | 1296.58 | 1297.05 | 0.07 |
| 1363.78 | 1363.6 | 1363.1 | 1363.98 | 1361.25 | 1363.14 | 0.08 |
| 1384.56 | 1384.8 | 1384.2 | 1384.25 | 1384.18 | 1384.38 | 0.02 |
| 1435.62 | 1436 | 1435.8 | 1436.79 | 1436.01 | 1436.04 | 0.03 |
| 1446.78 | 1446.2 | 1446.6 | 1446.78 | 1446.51 | 1446.56 | 0.02 |
| 1510.12 | 1510 | 1510.3 | 1510.36 | 1510.78 | 1510.31 | 0.02 |
| 1598.68 | 1598.8 | 1598.1 | 1598.7 | 1598.12 | 1598.48 | 0.02 |
| 1611.79 | 1611.8 | 1611.6 | 1612.14 | 1612.14 | 1611.89 | 0.02 |
| 2966.01 | 2966.1 | 2966.7 | 2968.05 | 2968.72 | 2967.12 | 0.04 |
| 2975.15 | 2976.1 | 2975.1 | 2975.65 | 2975.8 | 2975.57 | 0.01 |
| 3028.15 | 3029 | 3029.6 | 3030.17 | 3029.51 | 3029.29 | 0.03 |
| 3349.15 | 3350.2 | 3351.1 | 3351.01 | 3350.17 | 3350.32 | 0.02 |

Table S4. Five estrogen compounds determination via HPLC-FTIR for water sample (Yangtze River) after five estrogen compounds added

| Contaminants | BPA | DES | EE | E1 | E2 |
| --- | --- | --- | --- | --- | --- |
| Measure value  (mg/L) | 0.0029 | 0.0032 | 0.0028 | 0.0031 | 0.0029 |
| RSD% | 3 | 6 | 6 | 3 | 3 |
